# The Naples prognostic score serves as a predictor and prognostic indicator for cancer survivors in the community

**DOI:** 10.1186/s12885-024-12448-7

**Published:** 2024-06-06

**Authors:** Chaoqun Liang, Chao Zhang, Jun Song, Lin Yan, Yun Xiao, Nan Cheng, Han Wu, Xiaohong Chen, Jianming Yang

**Affiliations:** Department of Otolaryngology Head and Neck Surgery, The Second Affiliated Hospital of Anhui Medical University, Hefei, China

**Keywords:** Naples prognostic score (NPS), Cancer incidence, Cancer prognosis, NHANES (National Health and Nutrition Examination Survey), Community population

## Abstract

**Objective:**

Inflammation, malnutrition, and cancer are intricately interconnected. Despite this, only a few studies have delved into the relationship between inflammatory malnutrition and the risk of death among cancer survivors. This study aimed to specifically investigate the association between the categorically defined Naples prognostic score (NPS) and the prognosis of cancer survivors.

**Methods:**

Data from 42,582 participants in the National Health and Nutrition Examination Survey (NHANES, 1999–2018) were subjected to analysis. Naples prognostic scores (NPS) were computed based on serum albumin (ALB), total cholesterol (TC), neutrophil to lymphocyte ratio (NLR), and lymphocyte to monocyte ratio (LMR), and participants were stratified into three groups accordingly. Cancer status was ascertained through a self-administered questionnaire, while mortality data were sourced from the National Death Index up to December 31, 2019. Multiple logistic regression was employed to estimate the odds ratio (OR) with a 95% confidence interval (CI) between NPS and cancer prevalence within the U.S. community population. Kaplan-Meier survival analysis and the Log-rank test were utilized to compare survival disparities among the three groups. Additionally, Cox proportional regression was utilized to estimate the hazard ratio (HR) with a 95% CI.

**Results:**

The incidence of cancers was 9.86%. Among the participants, 8140 individuals (19.1%) were classified into Group 0 (NPS 0), 29,433 participants (69.1%) into Group 1 (NPS 1 or 2), and 5009 participants (11.8%) into Group 2 (NPS 3 or 4). After adjusting for confounding factors, the cancer prevalence for the highest NPS score yielded an odds ratio (OR) of 1.64 (95% CI: 1.36, 1.97) (P(_for trend_) < 0.05). In comparison to cancer survivors in Group 0, those with the highest NPS had adjusted hazard ratios (HRs) of 2.57 (95% CI: 1.73, 3.84) for all-cause mortality, 3.44 (95% CI: 1.64, 7.21) for cardiovascular mortality, 1.60 (95% CI: 1.01, 2.56) for cancer mortality, and 3.15 (95% CI: 1.74, 5.69) for other causes of mortality (All P(_for trend_) < 0.05). These associations remained consistent when stratified by age, sex, race, and body mass index.

**Conclusions:**

This study indicates that the Naples prognostic score (NPS), serving as a novel prognostic metric integrating inflammation and nutritional status, is closely linked to cancer prognosis within the general population.

**Supplementary Information:**

The online version contains supplementary material available at 10.1186/s12885-024-12448-7.

## Introduction

Cancer stands as one of the foremost causes of death worldwide, imposing substantial economic burdens on public health systems [1, 2]. Despite significant progress in cancer treatments in recent decades and notable enhancements in overall survival rates, the 5-year survival rate for cancer patients remains relatively modest, hovering around approximately 68.1% [3]. The long-term survival of cancer patients hinges on a variety of factors, encompassing the extent of tissue damage and inflammation, alongside psychological well-being [4]. Inflammation and malnutrition induced by cancer can instigate alterations in immune response and metabolism, thereby influencing cancer survival outcomes. Meanwhile, cancer survivors incur an additional annual healthcare expenditure of $3000–4500 on average compared to non-cancer patients, resulting in a greater burden on healthcare economies [5–7]. Therefore, identifying characteristic predictive indicators for cancer survivors and modifiable factors that can improve the long-term prognosis of cancer patients is crucial.

Recently, mounting evidence indicates that several nutrition and inflammation-related elements could function as robust predictive markers for individuals diagnosed with cancer. Notably, the neutrophil-to-lymphocyte ratio (NLR) and lymphocyte-to-monocyte ratio (LMR) have emerged as pivotal factors influencing the progression and prognosis of cancer patients [8, 9]. Indices related to nutrition, including the Prognostic Nutritional Index (PNI), Nutritional Risk Index (NRI), and Controlling Nutritional Status (CONUT), represent independent risk factors impacting overall survival (OS) in cancer patients. Nevertheless, establishing fixed optimal predictive cutoff values for continuous variables across studies presents a challenge, thereby complicating their application for general population assessment [10, 11]. Hence, there exists a requirement for a straightforward, universally defined indicator with consistent classification criteria across studies to facilitate population analysis.

The Naples Prognostic Score (NPS) is a novel scoring system originally utilized in evaluating the prognosis of colorectal cancer [12]. It is distinguished by its straightforward definition and consistent classification criteria across various studies [13–16]. The NPS comprises serum albumin (ALB), total cholesterol (TC), neutrophil-to-lymphocyte ratio (NLR), and lymphocyte-to-monocyte ratio (LMR), thereby offering a simultaneous reflection of the inflammatory and nutritional status of the body. Notably, NPS has been recognized as an independent prognostic factor in diverse hospitalized patients with organic diseases [17–21]. However, whether NPS can independently predict the association between cancer incidence and survival rates in community populations remains to be evaluated. Therefore, this study utilized data from the National Health and Nutrition Examination Survey (NHANES) spanning from 1999 to 2018 to explore the relationship between NPS and cancer incidence in the general population. Additionally, the study examined the correlation between NPS and mortality rates among cancer survivors. The goal is to furnish a straightforward predictive indicator for identifying cancer patients in the general population and to provide prognostic guidance for cancer survivors.

## Methods

### Study population and data collection

The study utilized data from the National Health and Nutrition Examination Survey (NHANES, 1999–2018), a nationally representative survey conducted by the National Center for Health Statistics [22]. NHANES aims to evaluate the nutritional and health status of the non-institutionalized population in the United States. All data are available for download from the official website (https://www.cdc.gov/nchs/nhanes) [22].

To ensure data reliability and completeness, the NHANES questionnaire collection process adheres to standardized and rigorously controlled procedures. The Computer-Assisted Personal Interviewing (CAPI) system, equipped with built-in consistency checks, plays a crucial role in minimizing data entry errors. Furthermore, the CAPI system incorporates an online help screen, offering valuable guidance to interviewers in precisely defining key terms in the survey questionnaire. This stringent quality assurance and control framework highlight our dedication to upholding high standards of data quality throughout NHANES, thereby bolstering the credibility and robustness of our study findings.

In this study, we analyzed NHANES data spanning from 1999 to 2018. Initially, participants under the age of 18 and those lacking data on cancer history assessment were excluded from the analysis. Subsequently, participants lacking assessment data for NPS (ALB, TC, NLR, and LMR), pregnant individuals, those with extreme energy intake (> 4200 or < 800 kcal/day for males; >3500 or < 500 kcal/day for females) [23], and those lacking weight information or follow-up information were also excluded.

### NPS assessment

(1)The NPS was defined based on ALB, TC, NLR, and LMR [12]. The optimal cut-off points for these indicators are determined using the MaxStat R package [24, 25], which identifies the values that maximize the log-rank statistic. This classification method has been employed since its initial reporting [26–28]. As described in previous literature, participants with serum albumin ≥ 40 g/L, TC > 180 mg/dL, NLR < 2.96, or LMR > 4.44 were assigned a score of 0, while those with serum albumin < 40 g/L, TC ≤ 180 mg/dL, NLR ≥ 2.96, or LMR ≤ 4.44 were assigned a score of 1. The NPS is calculated as the sum of scores for each of the four factors [12]. Patients were then categorized into three groups based on their NPS scores: group 0 (score of 0), group 1 (score of 1 or 2), and group 2 (score of 3 or 4).

### Cancer assessment

The NHANES study collected information on cancer history through a self-administered questionnaire [22]. Participants meeting the following two criteria were included: (1) Individuals who answered “Yes” to the question “Have you ever been told you had cancer or any type of malignant tumor?“; (2) Participants for whom a record of response to the question “What kind of cancer?” was available. Participants answering “No” to either question were used as the control group.

### Mortality assessment

The survival status of participants up to December 31, 2019, was determined by linking the study data with the National Death Index (NDI) [29]. This file provides the most recent linkage between selected National Center for Health Statistics(NCHS) surveys and the NDI. The International Classification of Diseases, Tenth Revision (ICD-10), was employed to delineate specific causes of death [30]. We scrutinized both all-cause mortality and specific causes of death, encompassing cardiovascular diseases (ICD-10: I00-I09, I11, I13, I20-I51) and malignant neoplasms (ICD-10: C00-C97). The baseline time for calculating survival time was defined as the time of NHANES data collection.

### Covariates

Baseline data on study participants were gathered through questionnaires and measurement data [22]. Self-reported variables such as age (in years), gender (male or female), education level (less than high school, high school, or higher than high school), race/ethnicity (non-Hispanic white, non-Hispanic black, Mexican American, or other race). Body mass index was measured by NHANES (BMI; <25.0, 25.0–29.9, or > 29.9 kg/m^2^). Poverty status was assessed using the poverty income ratio, calculated as the family poverty income ratio(PIR) divided by the poverty threshold determined based on family size according to guidelines from the U.S. Department of Health and Human Services. It was categorized as ≤ 1.0, 1.1-3.0, and > 3.0 [31, 32]. Individuals who smoked fewer than 100 cigarettes in their lifetime were classified as never smokers. Former smokers were defined as those who had smoked more than 100 cigarettes in their lifetime but had subsequently quit. Current smokers were defined as individuals who were currently smoking [33]. Alcohol consumption status was classified as follows: non-drinkers (individuals who reported no alcohol consumption in the past 12 months), light to moderate drinkers (men consuming < 3 drinks per day, women consuming < 2 drinks per day, or individuals engaging in binge drinking < 5 times in the past 30 days), or heavy drinkers (men consuming ≥ 4 drinks per day, women consuming ≥ 3 drinks per day, or individuals engaging in binge drinking ≥ 5 times in the past 30 days) [34, 35]. To ensure data accuracy, energy intake (kcal/day) was obtained through first 24-hour dietary recall interview [36].

### Statistical analysis

The NCHS analysis guidelines stipulate that all statistical analyses utilizing continuous NHANES data must incorporate the complex survey design to generate estimates representative of the civilian non-institutionalized population of the United States. Hence, the analysis includes sample weights as well as geographic clustering indicators (primary sampling units and strata) [37] .

Continuous variables are reported as weighted mean (standard error, SE), while categorical variables are presented as counts (weighted frequencies). Logistic regression analysis is utilized to compute adjusted odds ratios (OR) and 95% confidence intervals (CI) to examine the association between NPS and cancer incidence. Kaplan-Meier survival analysis and log-rank tests are employed to determine cumulative survival rates and compare them based on NPS score categorized into three groups (group 0: 0; group 1: 1–2; group 2: 3–4). Cox proportional hazards models are utilized to calculate adjusted hazard ratios (HR) and 95% CI for all-cause and cause-specific mortality rates among cancer survivors based on NPS.

Stratified analyses are performed based on age (< 45, ≥ 45 years) [38–40], gender (male, female), race (white, other), and BMI (< 30, ≥ 30) [41]. The significance of interactions is tested using p-values of the product terms between NPS and stratification variables. Trend tests for categorical variables are conducted based on NPS scores.

The statistical analysis will be carried out using R (version 4.3.3). Two-sided p-values less than 0.05 will be deemed statistically significant.

## Results

### Baseline characteristics of study participants

**Fig. 1:**
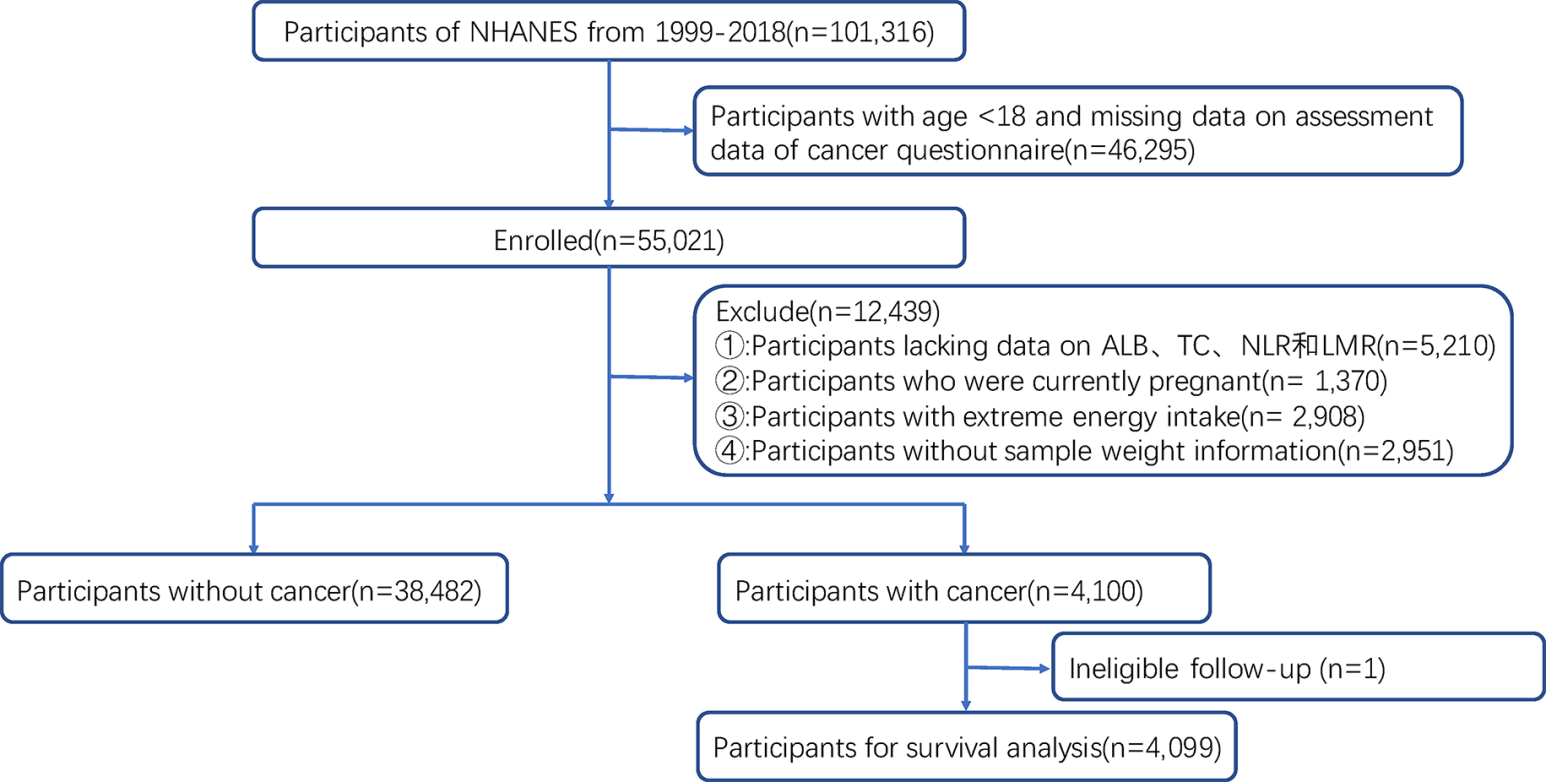
Participant flow diagram

Between 1999 and 2018, a total of 101,316 participants attended NHANES. Initially, participants under 18 years of age and those lacking cancer assessment data were excluded (*n* = 46,295). Subsequently, participants lacking NPS assessment data (*n* = 5,210), those who were pregnant during the survey period (*n* = 1,370), and those with excessive energy intake (*n* = 2,908) were also excluded. Participants without weight information, according to NHANES weighting guidelines [37], were further excluded (*n* = 2,951). Finally, 1 participant deemed unsuitable for follow-up was excluded from survival analysis. Therefore, a total of 42,582 participants were available for logistic regression, and 4,099 cancer survivors were included in survival analysis(Fig. 1).

Table 1 presents the baseline characteristics of three NPS score groups in NHANES from 1999 to 2018. The study population had a mean age of 47.74 (0.20) years, with males accounting for 47.45% and predominantly non-Hispanic whites (69.38%). Overall, the cancer incidence rate was 9.86%. Compared to Group 0, participants in Group 2 were more likely to be older non-Hispanic whites with lower levels of education and income, higher body mass index, and a higher prevalence of hypertension, with a lower prevalence of hyperlipidemia and diabetes. Additionally, the cancer incidence rate was significantly higher in Group 2 participants.

In a median follow-up period of 7.2 years, a total of 1,452 all-cause deaths were recorded, including 320 deaths attributed to heart disease and 430 deaths attributed to cancer(Table 2). Compared to adult cancer survivors, individuals who succumbed to various causes were more likely to be older Hispanic white males with higher NPS, lower levels of education and income, and lower energy intake. Additionally, participants who died from cancer exhibited a higher proportion of hypertension and diabetes.

### Association between NPS and cancer incidence

NPS was divided into three groups, with group 0 serving as the reference category, and its association with cancer incidence was evaluated (Table 3). The crude model’s odds ratios (ORs) with 95% confidence intervals (CIs) revealed a positive correlation between NPS and cancer incidence (1.36 (1.18, 1.56) for group 1; 2.20 (1.88, 2.57) for group 2). In the fully adjusted multivariable regression model, relative to group 0, the fully adjusted ORs (95% CIs) for groups 1 and 2 were 1.20 (1.03, 1.40) and 1.64 (1.36, 1.97), respectively. Across all models, the trend test indicated statistically significant associations (All P(_for trend_) < 0.05).

### Association between NPS and cancer survivor mortality

**Fig. 2:**
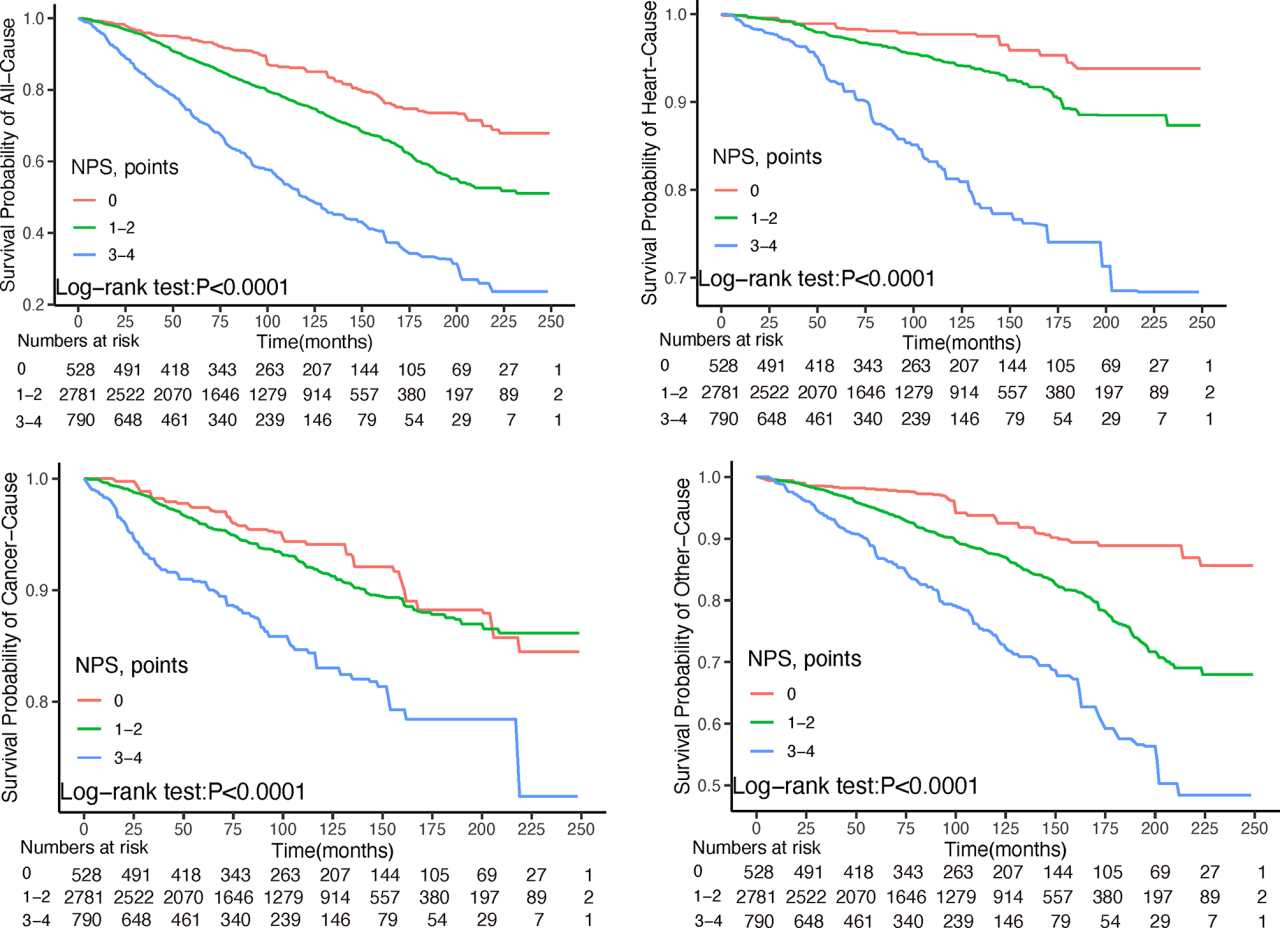
Kaplan‒Meier survival curve of mortality; (**A**) for all-cause mortality, (**B**) for cardiac mortality, (**C**) for cancer mortality, and (**D**) for other mortality

Firstly, Kaplan-Meier curves suggested significant differences in prognosis among cancer survivors across NPS groups (Fig. 2). The findings revealed that cancer survivors in Group 2 exhibited the highest risk of all-cause and other-cause mortality compared to the other two groups (log-rank test *P* < 0.0001). In the multivariable-adjusted models, hazard ratios (HRs) with 95% confidence intervals (CIs) indicated that, relative to Group 0, cancer survivors in Group 2 demonstrated elevated risks of mortality. The HRs (95% CIs) were 2.57 (1.73, 3.84) for all-cause mortality; 3.44 (1.64, 7.21) for cardiovascular mortality; 1.60 (1.01, 2.56) for cancer-specific mortality; and 3.15 (1.74, 5.69) for other-cause mortality (Table 4). In all models, the trend test revealed statistically significant associations (All P(_for trend_) < 0.05).

### Subgroup analysis

In the subgroup analysis (Table 5), when stratified by age (< 45, ≥ 45), gender (male, female), race (white, others), and BMI (< 30, ≥ 30), no significant differences in the impact of NPS on cancer survivor survival rates were observed among participants aged under 45 years. It is noteworthy that in the subgroup analysis, most results remained consistent with the main analysis trend. Regarding the mortality rates of cancer survivors, no significant interaction was found between each subgroup condition and NPS score (all P(_interaction_) > 0.05).

**Table 1:** Characteristics of adult participants in NHANES 1999–2018

Characteristics	Total	NPS, points			
		0	1-2	3-4	*P* value
Participants, N	42,582	8140	29,433	5009	
Age, years	47.74(0.20)	45.98(0.24)	47.38(0.21)	53.33(0.44)	< 0.0001
Energy intake, kcal/day	2051.95(6.21)	1987.84(11.40)	2075.00( 7.26)	2012.14(15.06)	< 0.0001
Age, %					< 0.0001
<45	17,085(45.08)	3448(47.14)	12,152(45.82)	1485(36.46)	
>=45	25,497(54.92)	4692(52.86)	17,281(54.18)	3524(63.54)	
Gender, %					< 0.0001
Female	21,990(52.55)	4939(62.59)	14,619(50.13)	2432(50.77)	
Male	20,592(47.45)	3201(37.41)	14,814(49.87)	2577(49.23)	
Race/ethnicity, %					< 0.0001
Other	7175(12.10)	1673(15.83)	4801(11.38)	701(10.26)	
Mexican American	7444( 8.01)	1748(10.16)	4984( 7.64)	712( 6.71)	
Non-Hispanic Black	8507(10.50)	1843(12.49)	5667( 9.89)	997(11.05)	
Non-Hispanic White	19,456(69.38)	2876(61.52)	13,981(71.10)	2599(71.98)	
Education level, %					< 0.0001
Below high school	11,211(16.51)	2210(16.96)	7641(16.17)	1360(18.04)	
High school	19,786(51.61)	3692(49.37)	13,637(51.73)	2457(55.18)	
Above high school	11,535(31.81)	2230(33.67)	8123(32.10)	1182(26.77)	
PIR, %					< 0.0001
<=1.0	7779(13.12)	1482(13.84)	5352(13.89)	945(15.67)	
1.1–3.0	16,448(33.19)	3016(34.83)	11,327(35.20)	2105(39.54)	
>3.0	14,842(46.96)	2912(51.33)	10,380(50.91)	1550(44.79)	
Body mass index, kg/m2, %					< 0.0001
<25.0	12,247(30.61)	2246(30.06)	8681(31.46)	1320(29.95)	
25.0–29.9	13,903(32.10)	2777(33.58)	9755(32.98)	1371(27.62)	
>=30.0	15,719(35.92)	3028(36.36)	10,574(35.56)	2117(42.43)	
Smoking, %					< 0.0001
Never smoker	23,074(53.82)	4671(55.46)	15,962(53.82)	2441(51.12)	
Former smoker	10,843(25.20)	1699(21.19)	7521(25.62)	1623(29.61)	
Current smoker	8637(20.94)	1767(23.34)	5928(20.55)	942(19.28)	
Alcohol, %					< 0.0001
Nondrinker	12,771(24.48)	2428(25.70)	8594(25.24)	1749(32.60)	
Low-to-moderate drinker	19,307(50.11)	3785(54.48)	13,440(53.73)	2082(49.09)	
Heavy drinker	7465(19.26)	1418(19.82)	5334(21.03)	713(18.31)	
Energy, %					< 0.0001
[500,1578]	14,199(30.21)	2828(32.75)	9579(29.34)	1792(31.53)	
(1578,2259]	14,196(33.81)	2784(35.00)	9730(33.44)	1682(34.20)	
(2259,4200]	14,187(35.97)	2528(32.25)	10,124(37.22)	1535(34.27)	
Hypertension, %					< 0.0001
No	24,107(62.09)	4954(66.03)	16,954(62.80)	2199(50.36)	
Yes	18,467(37.90)	3184(33.97)	12,474(37.20)	2809(49.64)	
Hyperlipidemia, %					< 0.0001
No	11,791(28.59)	1369(18.07)	8651(30.13)	1771(37.10)	
Yes	30,790(71.41)	6770(81.93)	20,782(69.87)	3238(62.90)	
DM, %					< 0.0001
No	7664(13.28)	1215(10.68)	4965(12.34)	1484(24.22)	
Yes	34,918(86.72)	6925(89.32)	24,468(87.66)	3525(75.78)	
Cancer survivors, %					< 0.0001
No	38,482(90.14)	7612(92.62)	26,651(90.23)	4219(85.10)	
Yes	4100( 9.86)	528( 7.38)	2782( 9.77)	790(14.90)	

Continuous variables are presented as weighted means (SE), while categorical variables are displayed as unweighted counts (weighted percentages). All estimates were adjusted for complex survey designs. Variables: N (study sample), PIR (poverty income ratio), DM (diabetes mellitus)

**Table 2:** Characteristics of adult with cancer in NHANES 1999–2018

Characteristics	Total (*N* = 4099)	All-cause mortality		Pvalue	
		Alive (*N* = 2647)	Deceased (*N* = 1452)		
Age, years	62.82(0.35)	59.53(0.39)	72.11(0.52)	< 0.0001	
Energy intake, kcal/day	1898.52(16.40)	1927.41(20.34)	1816.74(25.24)	< 0.001	
Age, %				< 0.0001	
<45	374(12.32)	355(15.90)	19( 2.17)		
>=45	3725(87.68)	2292(84.10)	1433(97.83)		
Gender, %				< 0.0001	
Female	2162(57.36)	1543(60.39)	619(48.78)		
Male	1937(42.64)	1104(39.61)	833(51.22)		
Race/ethnicity, %				0.02	
Other	374( 5.70)	315(6.42)	59(3.66)		
Mexican American	275( 2.30)	207(2.63)	68(1.36)		
Non-Hispanic Black	527( 5.14)	351(4.85)	176(5.94)		
Non-Hispanic White	2923(86.87)	1774(86.10)	1149(89.04)		
Education level, %				< 0.0001	
Below high school	908(14.31)	456(10.49)	452(25.13)		
High school	2016(56.65)	1512(62.66)	504(39.73)		
Above high school	1171(29.00)	676(26.85)	495(35.14)		
PIR, %				< 0.0001	
<=1.0	516( 9.26)	340( 9.14)	176(12.42)		
1.1–3.0	1636(31.25)	920(28.36)	716(49.06)		
>3.0	1616(52.14)	1174(62.49)	442(38.53)		
Body mass index, kg/m2, %				< 0.001	
<25.0	1144(28.94)	664(27.96)	480(34.05)		
25.0–29.9	1393(32.72)	894(33.02)	499(34.40)		
>=30.0	1466(36.37)	1055(39.02)	411(31.55)		
Smoking, %				< 0.0001	
Never smoker	1831(45.34)	1282(48.11)	549(37.49)		
Former smoker	1662(38.77)	944(35.70)	718(47.47)		
Current smoker	604(15.89)	419(16.19)	185(15.04)		
Alcohol, %				< 0.0001	
Nondrinker	1469(29.30)	760(25.05)	709(47.61)		
Low-to-moderate drinker	2029(54.85)	1414(62.65)	615(45.87)		
Heavy drinker	329(10.14)	259(12.30)	70( 6.51)		
Energy, %				0.003	
[500,1578]	1576(35.72)	954(33.74)	622(41.31)		
(1578,2259]	1475(37.22)	978(38.19)	497(34.48)		
(2259,4200]	1048(27.06)	715(28.06)	333(24.21)		
Hypertension, %				< 0.0001	
No	1440(41.16)	1078(46.71)	362(25.46)		
Yes	2659(58.84)	1569(53.29)	1090(74.54)		
Hyperlipidemia, %				0.64	
No	786(19.12)	492(18.90)	294(19.73)		
Yes	3313(80.88)	2155(81.10)	1158(80.27)		
DM, %				< 0.0001	
No	3041(78.68)	2026(81.08)	1015(71.91)		
Yes	1058(21.32)	621(18.92)	437(28.09)		
Cancer type				< 0.0001	
Other	532(13.14)	337(12.71)	195(14.54)		
Digestive system	361( 6.79)	187( 5.59)	174(10.28)		
Genital system	559(14.84)	450(17.25)	109( 8.24)		
Urinary system	789(12.57)	441(10.55)	348(18.47)		
Breast	604(14.51)	397(14.13)	207(15.79)		
Skin and soft tissue	1228(37.78)	815(39.78)	413(32.68)		
NPS, points				< 0.0001	
0	528(14.10)	417(16.01)	111( 8.69)		
1-2	2781(69.98)	1821(71.23)	960(66.45)		
3-4	790(15.92)	409(12.76)	381(24.86)		

Continuous variables are presented as weighted means (SE), while categorical variables are displayed as unweighted counts (weighted percentages). All estimates were adjusted for complex survey designs. Variables: N (study sample), PIR (poverty income ratio), DM (diabetes mellitus)

**Table 3:** ORs (95% CIs) of the prevalence of cancer according to the NPS in the NHANES 1999–2018 (*n* = 42,582)

Model	NPS, points		OR(95% CI)		P value		P trend
Crude	0		ref				<0.0001
	1-2		1.36(1.18,1.56)		<0.0001		
	3-4		2.20(1.88,2.57)		<0.0001		
Model 1	0		ref				<0.0001
	1-2		1.29(1.11,1.49)		0.001		
	3-4		1.90(1.61,2.24)		<0.0001		
Model 2	0		ref				<0.0001
	1-2		1.22(1.04,1.43)		0.01		
	3-4		1.75(1.44,2.12)		<0.0001		
Model 3	0		ref				<0.0001
	1-2		1.20(1.03,1.40)		0.02		
	3-4		1.64(1.36,1.97)		<0.0001		

Model 1 was adjusted for age (<45 or >=45), gender (male or female), and race (Non-Hispanic White, Non-Hispanic Black, Mexican American, or Other). Model 2 was adjusted for Model 1 plus education level (below high school, high school, or above high school), family income-to-poverty ratio (≤1.0, 1.1–3.0, or >3.0), smoking status (never smoker, former smoker, or current smoker), drinking status (nondrinker, low-to-moderate drinker, or heavy drinker), BMI (<25.0, 25.0–29.9, or >29.9), and energy intake levels (tertiles). Model 3 was adjusted for Model 2 plus Hypertension (Yes or No), Hyperlipidemia (Yes or No), and Diabetes (Yes or No)

**Table 4:** HRs (95% CIs) for all-cause and cause-specific mortality among cancer patients according to NPS

	NPS, points			Ptrend
	0	1-2	3-4	
All-cause mortality				
Crude	ref	1.77(1.35,2.32)	4.25(3.06,5.89)	<0.0001
Model 1	ref	1.58(1.18, 2.12)	3.52(2.49, 4.98)	<0.0001
Model 2	ref	1.53(1.12, 2.08)	2.93(2.02, 4.25)	<0.0001
Model 3	ref	1.47(1.06, 2.04)	2.57(1.73, 3.84)	<0.0001
Cardiovascular mortality				
Crude	ref	1.98(1.20, 3.27)	6.41(3.49,11.76)	<0.0001
Model 1	ref	1.67( 0.96, 2.91)	4.95(2.53,9.69)	<0.0001
Model 2	ref	1.67( 0.92, 3.01)	3.94(1.93,8.03)	<0.0001
Model 3	ref	1.60( 0.87, 2.94)	3.44(1.64,7.21)	<0.0001
Cancer mortality				
Crude	ref	1.16(0.78,1.72)	2.65(1.79,3.91)	<0.0001
Model 1	ref	1.01(0.68, 1.52)	2.11(1.43, 3.11)	<0.0001
Model 2	ref	0.97(0.63, 1.49)	1.79(1.16, 2.75)	0.003
Model 3	ref	0.95(0.61, 1.48)	1.60(1.01, 2.56)	0.021
Other-cause mortality				
Crude	ref	2.33(1.45,3.74)	4.95(2.90,8.46)	<0.0001
Model 1	ref	2.15(1.34, 3.46)	4.33(2.52, 7.43)	<0.0001
Model 2	ref	2.08(1.25, 3.48)	3.70(2.07, 6.61)	<0.0001
Model 3	ref	1.98(1.15,3.41)	3.15(1.74,5.69)	<0.0001

Model 1 was adjusted for age (<45 or >=45), gender (male or female), and race (Non-Hispanic White, Non-Hispanic Black, Mexican American, or Other). Model 2 was adjusted for Model 1 plus education level (below high school, high school, or above high school), family income-to-poverty ratio (≤1.0, 1.1–3.0, or >3.0), smoking status (never smoker, former smoker, or current smoker), drinking status (nondrinker, low-to-moderate drinker, or heavy drinker), BMI (<25.0, 25.0–29.9, or >29.9), and energy intake levels (tertiles). Model 3 was adjusted for Model 2 plus Hypertension (Yes or No), Hyperlipidemia (Yes or No), Diabetes (Yes or No), and Cancer type(skin and soft tissue, urinary system, breast, genital system, digestive system, and other)

**Table 5:** Subgroup analyses of the association of the frailty score with all-cause mortality among patients with cancer

Subgroup	NPS, points			*P*-trend	Per-point increment in NPS				
	0	1-2	3-4			*P*-interaction			
age						0.174			
>=45	ref	1.581(1.156,2.162)	2.798(1.893,4.135)	<0.0001	<0.0001				
<45	ref	0.479(0.134, 1.708)	1.980(0.193,20.353)	0.774	0.718				
gender						0.504			
Female	ref	1.330(0.881, 2.006)	2.758(1.594, 4.773)	<0.001	<0.0001				
Male	ref	1.902(1.205,3.003)	3.256(2.032,5.218)	<0.0001	<0.0001				
race						0.164			
Other	ref	0.782(0.412,1.483)	1.740(0.881,3.434)	0.179	0.004				
White	ref	1.665(1.160, 2.388)	2.956(1.915, 4.563)	<0.0001	<0.0001				
BMI						0.498			
<30	ref	1.685(1.213, 2.340)	2.904(1.935, 4.357)	<0.0001	<0.0001				
>=30	ref	1.210(0.696,2.102)	2.344(1.288,4.268)	<0.001	<0.001				

The model was adjusted for covariates, including age(<45 or >=45), gender(male or female), race(white or other), education level (below high school, high school, or above high school), family income-to-poverty ratio (≤1.0, 1.1–3.0, or >3.0), smoking status (never smoker, former smoker, or current smoker), drinking status (nondrinker, low-to-moderate drinker, or heavy drinker), BMI (<30, or >=30), energy intake levels (tertiles), Hypertension(Yes or No), Hyperlipidemia(Yes or No), Diabetes(Yes or No)

### Sensitivity analysis

In the sensitivity analysis, firstly, we divided NPS into 5 groups and re-conducted the aforementioned analyses. The results revealed that the positive correlation between NPS in 5 groups and cancer incidence rate, as well as the risk of mortality among cancer survivors, persisted (Table S1, Table S2). Secondly, we excluded participants who died within 2 years prior to follow-up and conducted survival analysis again. We confirmed the robustness of this relationship (Figure S1, Table S3).

## Discussion

This study utilized data from the NHANES database, and by appropriately weighting the data, our analysis outcomes accurately reflect the general population’s situation in the United States [37]. To our knowledge, this is the inaugural investigation exploring the association between NPS and cancer incidence rates. Our findings reveal that cancer survivors exhibit higher NPS scores compared to non-cancer participants. Following adjustments for numerous pertinent factors, we observed a significant positive correlation between NPS and the overall and specific cause mortality risk among cancer survivors. Given the simplicity of NPS calculation and the advantage of consistent standards across various studies [13–16], our research offers promising insights for the prospective application of NPS in tumor diagnosis and prognosis assessment.

The relationship between cancer and inflammation has been a subject of study since the 19th century. This consideration stems primarily from observational studies, which have noted that tumors frequently develop in areas of chronic inflammation. Moreover, biopsy samples of tumors commonly reveal the presence of inflammatory cells [42, 43]. Timely evaluation of the inflammatory status in cancer patients is crucial for comprehending disease progression and selecting suitable treatment strategies [43]. A substantial prospective cohort study using data from the UK Biobank database showcased a positive correlation between NLR and the risk of seven malignancies, while LMR exhibited a negative correlation. Notably, this correlation was particularly pronounced among patients diagnosed with malignancies within one year of recruitment [44]. Statistically, potential bodily infections and inflammatory responses are linked to approximately 15–20% of all cancer-related deaths globally [43]. Additionally, nutritional status plays a pivotal role in cancer progression, impacting the body’s oxidative stress levels and modifying tissue metabolism [45, 46]. Nutritional markers such as TC and ALB are closely intertwined with the advancement of malignant tumors [47, 48]. The interplay among cancer, inflammation, and nutrition underscores the significance of a comprehensive assessment of inflammation and nutrition in guiding tumor treatment [49]. For instance, studies have indicated that the integrated assessment of inflammation and nutrition is closely associated with predicting the efficacy of immunotherapy in malignant tumors [50–52].

NPS serves as a comprehensive reflection of overall inflammation, malnutrition, and survival across diverse conditions, demonstrating superior predictive performance compared to PNI, NRI, and CONUT scores [15, 53]. Moreover, as a categorical scoring system, NPS maintains consistency across studies, facilitating simpler comprehension for clinicians and patients alike, unlike other scoring systems [14, 15]. Beyond oncological diseases, NPS exhibits close associations with non-oncological conditions as well. Research has revealed links between NPS and hospitalization rates, as well as follow-up outcomes in patients with acute cardiovascular events [17, 19, 54, 55]. However, it’s noteworthy that prior studies have primarily focused on hospitalized patients, and the inherent vulnerability of this particular group may limit the broader generalization of this indicator. In our investigation, we observed that higher NPS levels correlate with increased cancer incidence and are closely linked with elevated overall and specific cause mortality rates (cardiovascular and cancer) among cancer survivors. Given the significant prevalence of comorbidity among cancer survivors [56], chronic cardiovascular and endocrine conditions can influence an individual’s inflammation and nutritional status [57, 58], potentially affecting the predictive capability of NPS for cancer disease. Addressing these concerns, our Model 3, after full adjustment, incorporated chronic disease states such as hypertension, hyperlipidemia, and diabetes. Encouragingly, the results remained robust despite these additional adjustments. This observation was further validated through various sensitivity analysis approaches. Although interaction analysis did not unveil statistically significant differences, it’s essential to acknowledge that the impact of NPS on cancer survivors was primarily concentrated among participants aged 45 and above. This can be attributed to three main factors. Firstly, cancer occurrence often exhibits time dependency, resulting in a potentially lower proportion of younger cancer survivors [59]. Secondly, aging individuals are more prone to inflammation and nutritional imbalances compared to their younger counterparts [60, 61]. Finally, individuals over the age of 45 are more likely to have cancer, cardiovascular disease, and serious endocrine diseases [38–40].

At the same time, it is worth noting that the cancer mortality rate was generally differentiated among the NPS groups compared to the superior differentiation of other deaths. However, the phenomenon may be widespread [62–67]. The possible explanation is that 969 cases of skin cancer, 619 cases of prostate cancer and 604 cases of breast cancer in our study, which account for 2192 of the total cancer patients. Non-melanoma skin cancers have a very low mortality rate [68], and their poor survival rates are often associated with non-cancer causes [69, 70]. In addition, breast and prostate cancer patients were also less likely to die from cancer, but more likely to die from non-cancer causes, such as heart disease, infections and suicide [71, 72].

Compared to previous studies, our research offers several notable advantages. Firstly, we leveraged a relatively large sample size with national representativeness and employed weighted strategies recommended by the NCHS, enabling us to elucidate the true relationship between NPS and both cancer incidence and mortality rates among cancer survivors [37]. Secondly, while previous studies were confined to hospital populations, limiting the applicability of the indicators, we expanded our investigation to encompass the entire community population in the United States, thereby enhancing the relevance and generalizability of our findings. Thirdly, the NPS index integrates overall inflammatory status and nutritional status, representing a significant advancement over single inflammatory or nutritional indicators in evaluating tumor progression. Lastly, we corroborated the association between NPS and cancer progression using various sensitivity analysis methods, ensuring robust analytical outcomes. The utilization of these methods underscores the substantial contribution of our study, offering valuable insights into cancer progression across diverse populations and healthcare settings.

This study also presents several limitations. Firstly, cancer-related data relied on self-reported information from participants, which could introduce recall bias. Nonetheless, NHANES implements standardized and stringent control procedures to ensure the reliability and completeness of the included data. Secondly, despite efforts to control for various potential confounders such as age, gender, and chronic disease status, there may still be unmeasured confounding variables that could impact the analysis. Thirdly, due to limited study data, the heterogeneity of cancer-related characteristics may not have been adequately assessed. To mitigate this potential bias, we adjusted for cancer categories in the analysis. Fourthly, given the observational nature of the study on cancer incidence correlation, causal relationships cannot be inferred. However, our study findings align with those of another prospective study from the UK Biobank [44]. Lastly, the study population primarily comprises participants from the US community, lacking data from economically underdeveloped countries, highlighting the need for further global research to validate the findings.

## Conclusion

The study reveals a noteworthy positive correlation between elevated NPS scores and cancer incidence. Moreover, higher NPS scores among cancer survivors are linked to heightened risks of both all-cause mortality and cause-specific mortality. These findings carry considerable clinical implications, indicating the potential utility of NPS in predicting clinical outcomes among cancer survivors and informing subsequent treatment strategies.

### Electronic supplementary material

Below is the link to the electronic supplementary material.

Supplementary Material 1

Supplementary Material 2

Supplementary Material 3

## Data Availability

The data used for all analyses are sourced from the public database NHANES, available at: [https://www.cdc.gov/nchs/nhanes/index.htm].

## Abbreviations

NPS: Naples Prognostic Score
NHANES: National Health and Nutrition Examination Survey
ALB: Albumin
TC: Total Cholesterol
NLR: Neutrophil to Lymphocyte ratio
LMR: Lymphocyte to Monocyte Ratio
OR: Odds Ratio
HR: Hazard Ratio
95%CI: 95%Confidence Interval
PNI: Nutritional Index
NRI: Nutritional Risk Index
CONUT: Controlling Nutritional Status
OS: Overall Survival
CAPI: Computer-Assisted Personal Interviewing
NDI: National Death Index
ICD-10: The International Classification of Diseases, Tenth Revision
NCHS: National Center for Health Statistics
SE: Standard Error
